# Supplementary material for: Aluminum Supplementation Mediates the Changes in Tea Plant Growth and Metabolism in Response to Calcium Stress
Source: Int J Mol Sci. 2023 Dec 30;25(1):530. doi: 10.3390/ijms25010530 (PMC10778998; doi:10.3390/ijms25010530)
Supplement: Supplementary file 1 [file ijms-25-00530-s001.zip › ijms-2763677-supplementary.pdf]

Aluminum Su pplementation Mediates the Changes in Tea Plant Growth and Metabolism in Response to Calcium Stress

Table S1 Effects of calcium and aluminum supply on metabolites content in tea shoots

Treatment	0.8Ca			6.5Ca			Ca	Al	Ca×Al
	0Al	0.4Al	1Al	0Al	0.4Al	1Al			
Caffeine	10.733±0.07b	10.72±0.087b	10.699±0.078b	10.937±0.026a	10.799±0.009b	10.691±0.031b	**	**	*
Catechin	10.892±0.051a	10.837±0.029a	10.866±0.025a	10.549±0.019b	10.602±0.007b	10.565±0.06b	**	ns	ns
Catechin gallate	6.449±0.052b	6.512±0.155a	6.477±0.015ab	6.414±0.045aba	6.422±0.012ab	6.345±0.037b	*	ns	ns
D-(-)-Fructose	9.019±0.03d	9.096±0.03c	9.057±0.053cd	9.251±0.026b	9.219±0.029b	9.349±0.029a	**	*	**
D-(-)-Quinic acid	9.051±0.079d	9.621±0.102b	9.299±0.042c	9.798±0.047a	9.583±0.012b	8.977±0.061d	**	**	**
DL-Arginine	10.584±0.08a	10.405±0.105b	10.489±0.084ab	10.56±0.04a	10.094±0.023c	10.215±0.037c	**	**	**
DL-Glutamine	9.839±0.291a	9.78±0.237a	9.354±0.269b	9.846±0.064a	9.752±0.022a	9.827±0.033a	ns	ns	ns
Epigallocatechin gallate	10.369±0.113ab	10.325±0.159ab	10.414±0.084a	10.183±0.036bc	10.447±0.013a	10.111±0.156c	*	ns	*
Glutamine	10.055±0.027a	9.957±0.116abc	9.644±0.055d	10.016±0.127ab	9.811±0.041c	9.872±0.053bc	ns	**	**
L-Aspartic acid	8.628±0.038a	8.487±0.024b	8.436±0.022b	8.474±0.043b	8.602±0.018a	8.465±0.018b	ns	**	**
L-Glutamic acid	9.509±0.277aba	9.654±0.144a	9.111±0.151c	9.322±0.119abc	9.596±0.2b	9.272±0.115bc	ns	**	ns
L-Phenylalanine	8.876±0.154ab	9.052±0.063a	8.9±0.116ab	9.014±0.107a	8.763±0.023b	8.756±0.133b	ns	ns	*
L-Threonine	8.557±0.072a	8.394±0.032a	8.154±0.103b	8.436±0.124a	8.508±0.131a	8.392±0.112a	ns	**	*
Rutin	8.179±0.093a	8.144±0.064a	7.831±0.085c	7.755±0.054c	8.009±0.053b	7.8±0.049c	**	**	**

Note: '*' means p-Value< 0.05, effect of the processing is significant; '**' means p-Value< 0.01, effect of the processing is extremely significant; 'ns' means no significant difference. Different letters above the bar indicate significant differences among treatments (P＜0.05).

Table S2 Effects of calcium and aluminum supply on metabolites content in tea roots

Treatment	0.8Ca			6.5Ca			Ca	Al	Ca×Al
	0Al	0.4Al	1Al	0Al	0.4Al	1Al			
Caffeine	9.217±0.083b	9.064±0.078c	9.686±0.067a	9.205±0.09b	9.68±0.07a	9.054±0.086c	ns	**	**
Catechin	8.48±0.022c	7.944±0.045d	7.984±0.104d	8.651±0.05b	8.016±0.047d	9.203±0.024a	**	**	**
Catechin gallate	6.962±0.08bc	6.914±0.068bc	6.897±0.079c	6.911±0.042bc	7.193±0.104a	7.049±0.072b	**	ns	**
D-(-)-Fructose	8.575±0.01a	8.59±0.008a	8.532±0.011b	8.409±0.044c	8.573±0.017a	8.302±0.021d	**	**	**
D-(-)-Quinic acid	8.264±0.061c	8.344±0.043b	8.15±0.042d	7.798±0.032f	8.524±0.021a	8.031±0.024e	**	**	**
DL-Arginine	10.138±0.015a	9.929±0.035c	9.952±0.027c	10.087±0.004b	9.778±0.014d	9.924±0.016c	**	**	**
DL-Glutamine	8.664±0.007b	8.854±0.014a	8.224±0.023d	8.154±0.016d	8.87±0.097a	8.532±0.019c	**	**	**
Epigallocatechin	7.98±0.029a	7.717±0.048c	7.013±0.044e	7.841±0.031b	7.697±0.015c	7.103±0.035d	ns	**	**
Epigallocatechin gallate	5.654±0.089cd	5.493±0.052d	6.052±0.172b	5.838±0.171bc	6.428±0.086a	5.84±0.135bc	**	*	**
Glutamine	9.818±0.111a	9.753±0.024a	9.369±0.019b	9.356±0.124b	9.856±0.043a	9.389±0.024b	**	**	**
L-Aspartic acid	8.767±0.06a	8.686±0.037b	8.607±0.004c	8.691±0.03b	8.691±0.022b	8.37±0.008d	**	**	**
L-Glutamic acid	9.107±0.048a	8.998±0.044b	8.903±0.005cd	8.951±0.066bc	9.025±0.052b	8.853±0.025d	*	**	*
L-Phenylalanine	9.012±0.047ab	9.019±0.033ab	9.118±0.085a	8.862±0.244b	9.169±0.081a	8.802±0.073b	ns	ns	*
L-Threonine	8.493±0.04a	8.475±0.096ab	8.455±0.086ab	8.462±0.012ab	8.5±0.061a	8.328±0.116b	ns	ns	ns
Rutin	6.646±0.099ab	6.42±0.083bc	6.594±0.159ab	6.582±0.145ab	6.753±0.133a	6.265±0.111c	ns	ns	**

Note: '*' means p-Value< 0.05, effect of the processing is significant; '**' means p-Value< 0.01, effect of the processing is extremely significant; 'ns' means no significant difference. Different letters above the bar indicate significant differences among treatments (P＜0.05).
